# Functional Alterations in Neural Substrates of Geometric Reasoning in Adults with High-Functioning Autism

**DOI:** 10.1371/journal.pone.0043220

**Published:** 2012-08-17

**Authors:** Takashi Yamada, Haruhisa Ohta, Hiromi Watanabe, Chieko Kanai, Masayuki Tani, Taisei Ohno, Yuko Takayama, Akira Iwanami, Nobumasa Kato, Ryuichiro Hashimoto

**Affiliations:** 1 Department of Psychiatry, Showa University School of Medicine, Tokyo, Japan; 2 Japan Science and Technology Agency, CREST, Tokyo, Japan; United Graduate School of Child Development, Osaka University, Japan

## Abstract

Individuals with autism spectrum condition (ASC) are known to excel in some perceptual cognitive tasks, but such developed functions have been often regarded as “islets of abilities” that do not significantly contribute to broader intellectual capacities. However, recent behavioral studies have reported that individuals with ASC have advantages for performing Raven's (Standard) Progressive Matrices (RPM/RSPM), a standard neuropsychological test for general fluid intelligence, raising the possibility that ASC′s cognitive strength can be utilized for more general purposes like novel problem solving. Here, the brain activity of 25 adults with high-functioning ASC and 26 matched normal controls (NC) was measured using functional magnetic resonance imaging (fMRI) to examine neural substrates of geometric reasoning during the engagement of a modified version of the RSPM test. Among the frontal and parietal brain regions involved in fluid intelligence, ASC showed larger activation in the left lateral occipitotemporal cortex (LOTC) during an analytic condition with moderate difficulty than NC. Activation in the left LOTC and ventrolateral prefrontal cortex (VLPFC) increased with task difficulty in NC, whereas such modulation of activity was absent in ASC. Furthermore, functional connectivity analysis revealed a significant reduction of activation coupling between the left inferior parietal cortex and the right anterior prefrontal cortex during both figural and analytic conditions in ASC. These results indicate altered pattern of functional specialization and integration in the neural system for geometric reasoning in ASC, which may explain its atypical cognitive pattern, including performance on the Raven's Matrices test.

## Introduction

The exact nature of autistic cognition and intelligence has been a subject of extensive controversy. Individuals with autism spectrum condition (ASC) sometimes perform better than neurotypical individuals on certain tasks, including visual search and the Block Design and Object Assembly subtests of the Wechsler Intelligence Scale (WAIS) [1], [2]. However, these strengths of autistic ability are often thought to be limited to domain-specific low-level perceptual functions and the ASC′s enhanced sensation of the local information to arise at the sacrifice of high-level integrative and hierarchical processes involving diverse types of computation and information [3], [4]. Yet, such characterization of ASC′s intelligence is not directly compatible with a recent finding showing an unexpectedly high performance of autistic individuals on the Raven's Progressive Matrices (RPM) test, one of the most widely-used neuropsychological tests for general fluid intelligence. In the RPM, adults and children with ASC have been reported to score 30 percentile points higher on average than scores expected based on their performance on the Wechsler scales of intelligence [5]. Moreover, children with Asperger's syndrome have been shown to outperform typically developing children matched for full-scale IQ on the WISC in the Raven's Standard Progressive Matrices (RSPM) test [6]. Because the Raven's Matrices tests critically involve various types of high-level processes including abstraction, rule inference, and hierarchical goal management [7], [8], these findings suggest that perceptual and cognitive strengths of ASC are not limited to simple low-level tasks but are utilized for solving novel problems.

The RSPM test consists of figures of geometric matrices that can be categorized into two major classes of “figural” and “analytic” items [9], [10]. “Figural” items are often characterized as “Gestalt reasoning,” which requires mostly visuospatial analysis with minimal analytic/analogical reasoning. In contrast, “analytic” problems require abstract “analogical reasoning” in addition to figural processing [7], [9]. Given that analytic items occupy the majority of the RSPM set (41 analytic items out of 60) and that figural items are relatively simple for high-functioning individuals, the analytic items constitute a core component of the task that critically contributes to the RPM advantage of high-functioning ASC.

Neural substrates for general intelligence have been examined in healthy adult individuals by using functional imaging techniques [7], [8], [11]–[14]. Although exact locations of brain activation may vary depending on task parameters, a recent meta-analysis of the literature emphasized that a set of the frontoparietal regions, including the inferior parietal cortex (IPC), the ventrolateral prefrontal cortex (VLPFC) and the dorsolateral prefrontal cortex, is consistently involved. This observation led to the proposal of the Parietal-Frontal Integration Theory (P-FIT) of intelligence which states that intelligence is subserved by the large-scale network of the prefrontal and parietal regions [15]. Although the functional role of each individual region of the network remains unclear, a recent functional magnetic resonance imaging (fMRI) study reported a dichotomy of the fluid intelligence network such that activity in the right lateralized network was modulated by rule complexity, whereas activity in the left anterior lateral occipital cortex (LOC) and VLPFC was sensitive to analogical reasoning demand [16].

In the present study, brain activity of high-functioning ASC participants during the RSPM task was measured using fMRI to examine functional properties of their brain network of geometric analogical reasoning. A previous fMRI study reported that adult individuals with ASC showed enhanced RSPM task-related activity in the extrastriate visual cortex and reduced activity in the lateral frontal regions including the precentral gyrus [17]. Whereas that study combined all the figural and analytic items for analysis, the first purpose of the present experiment was to identify neural bases of ASC for high-level cognitive processes critical for analogical reasoning, including abstraction, rule inference, and hierarchical goal management. We aimed to isolate activation for these processes by directly comparing between the analytical and figural items based on the view that cognitive components for the analytical items subsume those for the figural items [7]. From previous evidence of enhanced visual perception as well as increased activation in the occipital and lateral occipitotemporal cortex (LOTC) during intelligence tasks in ASC [17]–[19], we hypothesized that ASC would show enhanced activity in the localized posterior regions for geometric analogical reasoning. In addition to localized brain activity, we also examined activation coupling between regions in the large-scale network of fluid intelligence [15]. Because analysis of functional connectivity does not technically require comparison between task conditions, we aimed to examine possible alterations of functional connectivity during solving the figural and analytical items. From the previous findings that the ASC brain can be characterized by scant long-range connections in the presence of excessive local connections [20]–[22], we hypothesized that ASC would show reduced long-range inter-regional activity coupling either between the anterior and posterior regions or inter-hemispheric regions.

## Materials and Methods

### Participants

**Figure 1:**
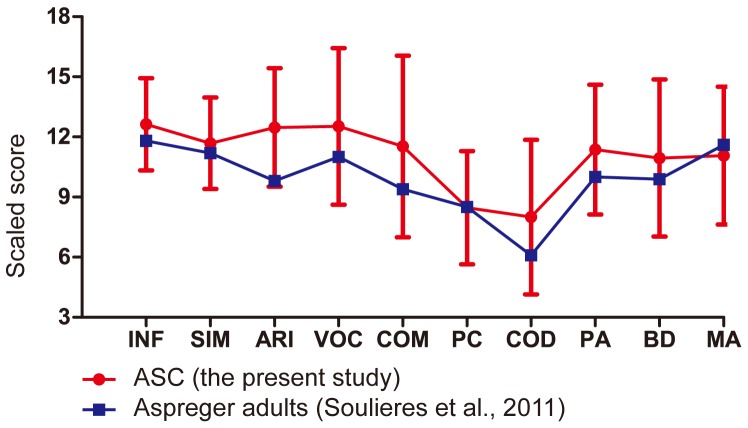
Wechsler subtest profiles in our study and those in a previous study [**24**]. The profile of the adult autism spectrum condition (ASC) participants in the present study is shown in red and that of the Asperger adults in a previous study (adapted from Soulieres et al., 2011) in blue. Error bars indicate the standard deviation of mean. INF, Information; SIM, Similarity; ARI, Arithmetic; VOC, Vocabulary; COM, Comprehension; PC, Picture Completion; COD, Digit Symbol-Coding; PA, Picture Arrangement; BD, Block Design; MA, Matrix.

Twenty-five high-functioning adults with ASC and 26 normal control (NC) adults participated in this study (Table 1). The two groups were matched for age and gender ratios. Handedness was assessed using the Edinburgh Handedness Inventory [23]. The IQ scores of the ASC participants were determined using either the Wechsler Adult Intelligence Scale–Third Edition (WAIS-III) or the WAIS–Revised. All participants with ASC scored higher than 80 for full-scale IQ (minimum, 83), which qualified them as high-functioning. Verbal IQ was significantly higher than performance IQ (*p*<0.001). Figure 1 illustrates the WAIS subtest profile of our ASC population, which was similar to that of adults with Asperger's syndrome regarding peaks on the Information, Vocabulary, and Matrix Reasoning subtests, a trough in the Digit Symbol-Coding, and no obvious advantage in the Block Design reported previously [24]. This pattern of the WAIS subtest was also consistent with our previous study of high-functioning ASC participants [25]. The IQ scores of the NC participants were estimated using a Japanese version of the National Adult Reading Test (JART) [26]. No significant difference was observed between the full-scale IQ scores of the ASC group and the JART-estimated IQ scores of the NC group (*p*>0.95, Table 1). All the participants also completed the Japanese version of the Autism-Spectrum Quotient (AQ) test [27].

**Table 1:** Demographic data and rating scale scores.

Subject	NC	ASC
Number	26(4 female)	25(3 female)
Age	32.2±7.7	30.7±7.78
Handedness a	86.4±39.9	55.7±72.4
WAIS full scale IQ b	103.9±11.4	106.9±15.9
WAIS verbal IQ b	105.5±11.7	113.5±15.2
WAIS performance IQ b	101.2±12.1	97.1±16.7
JART estimated IQ^c^	106.7±9.4	
AQ: total score d, †	16±6.4	35.7±5.6

Mean±standard deviation.

NC: Normal Control, ASC: Autism Spectrum Condition, WAIS: Wechsler Adult Intelligence Scale, JART: Japanese version of National Adult Reading Test, AQ: Autism Spectrum Quotient.

a Assessed by the Edinburgh handedness inventory (Oldfield, 1971).

Data was collected from 21NC and 22 ASC participants.

b WAIS- III or -R was performed by 15 NC and 25 ASC participants.

c The JART score was collected from all NC participants.

d The AQ was collected from 19 NC and 25 ASC participants.

† A significant difference between NC and ASC (*p*<0.001).

ASC participants were recruited from outpatient units of the Karasuyama Hospital, Tokyo, Japan. The inclusion criteria were age between 18 and 50 years, no current use of psychotropic medication, and a formal diagnosis of pervasive developmental disorder (PDD) based on the Diagnostic and Statistical Manual of Mental Disorders, Fourth Edition (DSM-IV). The exclusion criteria included a history of electroconvulsive therapy, alcohol or other drug abuse or dependence, or any neurologic illness affecting the central nervous system. PDD was diagnosed by a team of three experienced psychiatrists and one clinical psychologist on the basis of two detailed patient interviews of the patients regarding their development and behavior from infancy through adolescence [(1) developmental history; (2) present illness; (3) past history] and family history. The interviews were conducted independently by one psychiatrist and the clinical psychologist in the team. The patients were also asked to bring suitable informants who had known them in early childhood. At the end of the clinical interview, the psychiatrist diagnosed the patients according to the DSM-IV diagnostic criteria for PDD based on the consensus of the psychiatrists and the clinical psychologist. The diagnostic process required approximately 3 hours. These assessments resulted in all of the participants in the ASC group receiving clinical diagnoses of Asperger's disorder (n = 11), high-functioning autism (n = 11), or pervasive developmental disorder not otherwise specified (n = 3). Two of the psychiatrists and the psychologist confirmed that none of the participants met the DSM-IV criteria for any other psychiatric disorder (e.g., mood disorder, schizophrenia, anxiety disorder, or substance-related disorder). In addition, none of the NC subjects reported any severe medical problem or any neurological or psychiatric history. Moreover, the Mini-International Neuropsychiatric Interview was used to confirm that none of the normal controls met the diagnostic criteria for any psychiatric disorder. All of the participants had normal or corrected-to-normal vision. All the procedures of this study, including the method of obtaining consent, were approved by the Ethics Committee of the Faculty of Medicine of Showa University in accordance with the Declaration of Helsinki. Written informed consent was obtained from each participant after a full explanation of the purpose of the study was provided. Because participants were all high-functioning (IQ>80) adults without any other comorbidities, they were able to fully understand the content and nature of the study. Guardians' oral consents were not either documented or recorded because every patient was judged to possess the full ability to give consent on his/her own by his/her primary doctor (TY, HO, WH, or NK). Any concern regarding the possibility of reduced capacity to consent on his/her own was not voiced by either the ethics committee or patients' primary doctors. Every participant was assigned arbitrary identification number for this study such that all the data, including imaging, behavioral, and demographic data, was analyzed anonymously.

### Task and experimental setup

**Figure 2:**
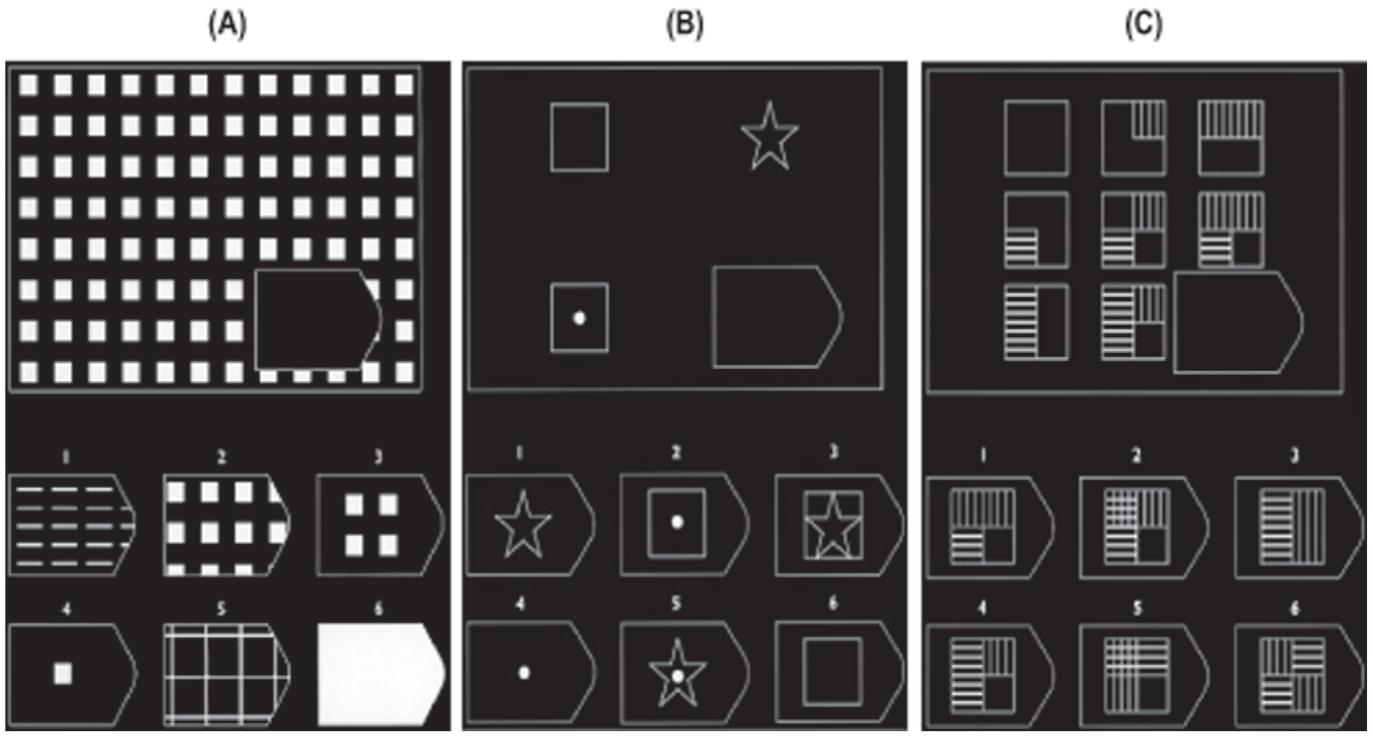
Sample stimuli for the Raven's Progressive Matrices problems. (A) “Figural” condition, (B) “easy analytic” condition, and (C) “difficult analytic” condition. Participants were asked to select one out of the six alternatives by pressing a button.

We measured brain activity during “figural,” “easy analytic,” and “difficult analytic” conditions using selected items of the RSPM set (Figure 2). The classification of “figural” and “analytic (analogical reasoning)” items was based on previous studies [9], [10]. Within “analytic/analogical reasoning” items, we used B8–C6 for the “easy” analytic condition of moderate difficulty and C7–D6 for the “difficult” analytic condition of high difficulty. We did not use items between D7 and E12 because those items usually take significantly longer than one fMRI task block length of 30 seconds (see next paragraph) according to our pilot experiment for healthy adults who had IQs comparable with the present population. By selecting relatively easy items, we also aimed to reduce general task difficulty following the previous proposal that behavioral performances under fMRI sessions should be comparable between groups to exclude confounds for the interpretation of group difference of fMRI results [28], [29]. Further, we modified each “figural” item and increased the total number of items by changing the orientation and rearranging the array of the six options. Although each original problem had either six or eight response alternatives, we removed two in the problems with eight alternatives to reduce possible confusion in button pressing in the restricted space inside the MRI system.

The RSPM task was performed in a block design. Blocks for easy and difficult analytic items alternated twice while blocks for figural served as a baseline, which resulted in nine blocks in total. Participants were instructed to select an answer among six response alternatives by pressing a button and were encouraged to deliberate thoroughly until they were reasonably certain about their answers. Duration of each task block was 30 seconds except for the last baseline block of 32 seconds. Participants proceeded in a self-paced manner during each block. Because the duration of each block was fixed, the last trial was usually terminated without button pressing. During a single fMRI session, NC and ASC individuals performed 27.84±8.27 (mean±standard deviation) and 27.70±8.53 trials for the figural condition, 6.27±2.42 and 6.88±2.05 trials for the easy analytic condition, and 2.61±1.60 and 3.45±2.18 trials for the difficult analytic condition, respectively. No significant group difference was observed between any of the conditions (*p* = 0.95 in figural; *p* = 0.35 in easy analytic), but ASC tended to proceed faster than NC in the difficult analytic condition (*p* = 0.12).

The RSPM figures were projected on an fMRI-compatible screen that was placed at the participant's feet. The participants were immobilized with padding and watched the screen through a mirror mounted on the headcoil. The subjects' responses were collected via a fiber-optic button box that was held by the participant while in the magnet. The button box was connected to an interface for the PC that allowed for the collection of response time and accuracy data (Current Designs, Philadelphia, PA). The presentation of the stimuli and collection of the responses were performed using Neurobehavioral System's Presentation software (Neurobehavioral Systems, Albany, CA).

### Data acquisition

Magnetic resonance (MR) images were acquired using a 1.5 T scanner with an 8-channel phased-array head coil (Signa Horizon; General Electric Medical Systems, Milwaukee, WI). Functional data were acquired using an echo planar imaging (EPI) sequence (27 axial slices, voxel size = 3.43×3.43×4 mm, TR = 2000 ms, TE = 40 ms, flip angle = 90°). During a single run, 136 volumes were acquired. A high-resolution structural T1 image was also acquired (gradient-echo; TR = 25 ms, TE = 9.2 ms, matrix size = 256×256×175, flip angle = 90°, voxel size = 0.975×0.975×1.4 mm) to allow for anatomical localization of activation.

### fMRI data preprocessing and analysis

Preprocessing and statistics of fMRI time-series data were performed using Statistical Parametric Mapping software (SPM5) (Wellcome Department of Cognitive Neurology, London, UK) running on MATLAB version R2009b (The Mathworks, Inc., Natick, MA). Functional images were realigned, normalized into the standard Montreal Neurological Institute (MNI) template, and smoothed using an 8 mm FWHM Gaussian smoothing kernel. The images were resampled to a resolution of 2×2×2 mm when they were normalized. In a first-level general linear model analysis for individual participants, task-related activation was modeled by boxcar functions for easy and difficult analytic blocks, each of which was convolved with the hemodynamic function. For each participant, the contrast images of easy vs. baseline, difficult vs. baseline, and analytic (easy+difficult) vs. baseline were generated. The contrast images were fed into a random-effects second-level analysis. Statistical threshold was set to *p*<0.001 at the voxel level, uncorrected for multiple comparisons. The spatial threshold was set at 72 contiguous voxels, as determined by 10000 Monte Carlo simulations using the AlphaSim program to achieve a family-wise error rate of *p* = 0.05 at the cluster level.

The comparison of analytic (easy+difficult) vs. baseline produced reliable clusters of activation in regions of the network of general fluid intelligence consisting of the bilateral VLPFC, premotor cortex (PMC), and IPC, as well as the area around the left LOTC [15], [16]. For ROI analysis, the voxel coordinate with the highest t-value in each cluster was first identified by comparing analytic (easy+difficult) vs. baseline in the combined analysis of the NC and ASC groups. The estimate of activation (beta-value) was then extracted during the easy and difficult conditions from 27 voxels centering that voxel in each individual. Three-way ANOVA was performed with the between-subject factors of Group and Region and the within-subject factor of Condition for each ROI.

Motivated by the P-FIT model of intelligence [15], we then examined the activation coupling between the parietal and frontal regions by performing a functional connectivity analysis. Among the regions used for the aforementioned ROI analysis, we selected the bilateral IPC, bilateral PMC, and right VLPFC as seed regions because ROI analysis did not reveal any significant effect related to group in the activation magnitude for these regions, which qualifies them as unbiased seed regions for functional connectivity analysis. First, time-series data of each voxel in the individual brain were regressed out for the six parameters of head movement, mean signal intensity in the whole scanning range, and mean intensity in the white matter and cerebrospinal fluid (CSF) regions as previously described [30]. Then, reference time-series of each ROI was created by collapsing the entire time-series (136 data points) of the same 27 voxels used for the ROI analysis. Next, we calculated the Pearson's correlation coefficient between a ROI time-series and the time-series of each voxel in the whole brain to create an r-map for each individual participant, which was further transformed to a z-map to be fed into the second-level analysis. We first performed a one-sample t-test using the z-maps of all the participants (*p*<0.05, uncorrected) and then used the resulting map as an inclusive mask for the subsequent two-sample t-test for between-group comparison to exclude voxels with negative correlations. For the between-group comparison, statistical threshold was set to uncorrected *p*<0.001 at the voxel level, and spatial extent threshold was set at *k*>72 voxels to satisfy the criteria of FWE-corrected *p*<0.05 at the cluster level.

When any significant voxel cluster was found in the group comparison, we next examined activation coupling at each of figural (baseline), easy analytic, and difficult analytic conditions separately. For each condition, we first created r- and z-maps in each participant following the same procedures described above except that the number of data points was 60 (12 points×5 blocks), 24 (12 points×2 blocks), and 24 (12 points×2 blocks) for figural (baseline), easy analytical, and difficult analytical conditions, respectively. The first three data points were removed to exclude signal fluctuations due to block transition [31], [32], which resulted in 12 data points in each block. We then extracted mean z-values from the cube of 27 voxels centering the voxel showing the highest t-value within a significant voxel cluster identified in the group comparison. Finally, we performed a two-way ANOVA of Condition (figural, easy analytical, and difficult analytical) x Group (NC and ASC) for that cluster.

## Results

### Behavioral data

The mean number of correct answers and response times (RT) are shown in Table 2. Due to a technical problem, the behavioral data of one ASC participant could not be included. A two-way ANOVA (Group×Condition) on the number of correct answers showed a highly significant main effect of Condition (*F* = 179.56, *p*<0.001), whereas no significant main effect of Group (*F* = 0.95, *p* = 0.34) or interaction (*F* = 0.71, *p* = 0.40) was found. Post-hoc tests revealed significant differences in all of the three comparisons of baseline vs. easy analytic, baseline vs. difficult analytic, and easy vs. difficult analytic (all *p*<0.05). A highly significant main effect of Condition was also found on RT (*F* = 91.92, *p*<0.001), whereas no significant main effect of Group (*F* = 0.11, *p* = 0.75) or interaction (*F* = 1.60, *p* = 0.21) was found. Post-hoc tests again revealed significant differences in all of the three comparisons (all *p*<0.05). No significant main effect of Group on either the number of correct answers or RT indicates that both NC and ASC participants performed the task comparably. Significant differences of accuracy and RT among the three conditions confirm that the task difficulty was manipulated as expected.

**Table 2:** Behavioral results.

	NC	ASC
Baseline		
Number of correct answers	24.08±8.40	23.92±8.31
Response time (s)	5.02±1.95	4.90±1.59
Easy analytic		
Number of correct answers	4.35±2.45	5.17±2.16
Response time (s)	8.27±3.80	7.21±2.45
Difficult analytic		
Number of correct answers	0.88±0.95	0.83±0.87
Response time (s)	14.74±5.14	13.46±5.47

Mean±standard deviation.

NC: Normal Control, ASC: Autism Spectrum Condition.

No significant main effect of Group either on the number of correct answers (*F* = 0.95, *p* = 0.34) or on response time (*F* = 0.11, *p* = 0.75).

### Voxel-based whole-brain analyses

**Figure 3:**
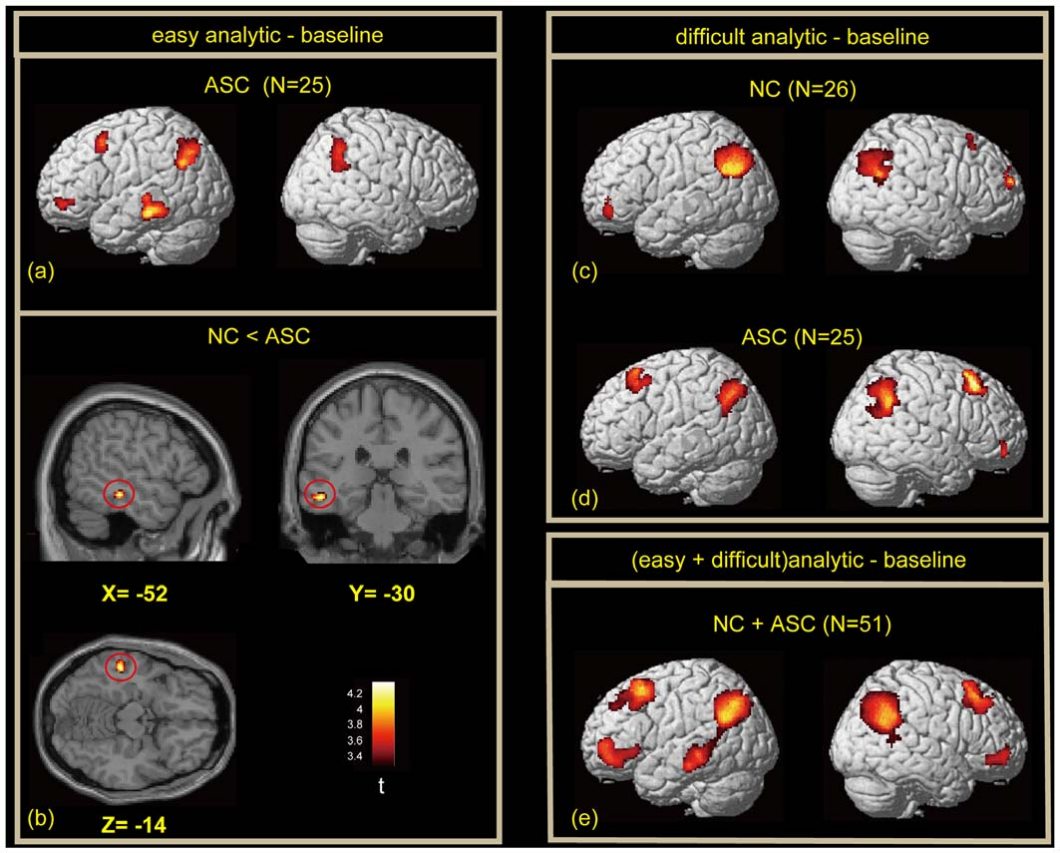
Lateral surface rendering of activation during the easy, difficult, and (easy+difficult) analytic conditions. (a) Participants with autism spectrum condition (ASC) during the easy analytic condition. Normal control (NC) showed no significant activation during the easy analytic condition. (b) Direct comparison between NC and ASC (NC<ASC) during easy analytic condition. (c) NC during the difficult analytic condition. (d) ASC during the difficult analytic condition. (e) Combined analysis of the two groups during the two analytic conditions. Statistical threshold was set at *p*<0.001, uncorrected for multiple comparisons. Spatial extent threshold was *k*>72 voxels.

In the contrast of easy analytic vs. baseline, significant activation was observed in the bilateral IPC, left premotor cortex (PMC) and VLPFC, and a part of the left LOTC (junction of the left inferior temporal cortex and anterior LOC) in ASC, whereas NC showed no significant brain activation (Figure 3a and Table 3). Direct comparison between groups revealed significantly enhanced activation in the left LOTC in ASC (Figure 3b). Moreover, no areas showed significantly larger activation in NC than in ASC. In the difficult analytic condition, prominent activation was identified in the bilateral IPC and frontal regions in both groups (Figure 3c, 3d and Table 4). No significant cluster was identified in the direct comparison between groups.

**Table 3:** Significant activity during easy analytic condition.

	Left					Right				
Region label	x	y	z	z value	Cluster size	x	y	z	z value	Cluster size
A: NC No significant voxels										
B: ASC										
LOTC	−60	−32	−14	4.83	523					
IPC	−48	−66	42	4.22	556	54	−54	50	3.86	354
PMC	−44	14	54	4.17	187					
VLPFC	−42	50	−6	4.12	106					
C: NC>ASC	No significant voxels									
D: NC<ASC										
LOTC	−52	−30	−14	3.99	85					

*p*<0.001, uncorrected for multiple comparison at the voxel level.

Extent threshold: *k*>72 voxels. The coordinates are in the MNI space.

NC: Normal Control, ASC: Autism Spectrum Condition, LOTC: lateral occipitotemporal cortex.

IPC: inferior parietal cortex, PMC: premotor cortex, VLPFC: ventrolateral prefrontal cortex.

NC showed no significant brain activation during the easy analytic condition.

No areas showed larger activation for NC than ASC.

**Table 4:** Significant activity during difficult analytic condition.

	Left					Right				
Region label	x	y	z	z value	Cluster size	x	y	z	z value	Cluster size
A: NC										
IPC	−58	−56	42	5.18	1301	52	−74	32	5.34	720
VLPFC	−42	48	−8	4.08	121	22	60	20	3.69	223
PMC						24	24	62	3.61	79
Precuneus	−6	−62	34	3.99	236					
B: ASC										
IPC	−46	−70	48	4.81	574	56	−58	48	4.65	876
PMC	−20	20	62	4.02	447	32	26	52	4.30	777
VLPFC						32	52	−14	3.91	92
C: NC>ASC	No significant voxels									
D: NC<ASC	No significant voxels									

*p*<0.001, uncorrected for multiple comparison at the voxel level.

Extent threshold: *k*>72 voxels. Coordinates are in the MNI space.

NC: Normal Control, ASC: Autism Spectrum Condition, IPC: inferior parietal cortex.

VLPFC: ventrolateral prefrontal cortex, PMC: premotor cortex.

No significant activation was identified by direct comparisons between the groups.

### ROI analyses

**Figure 4:**
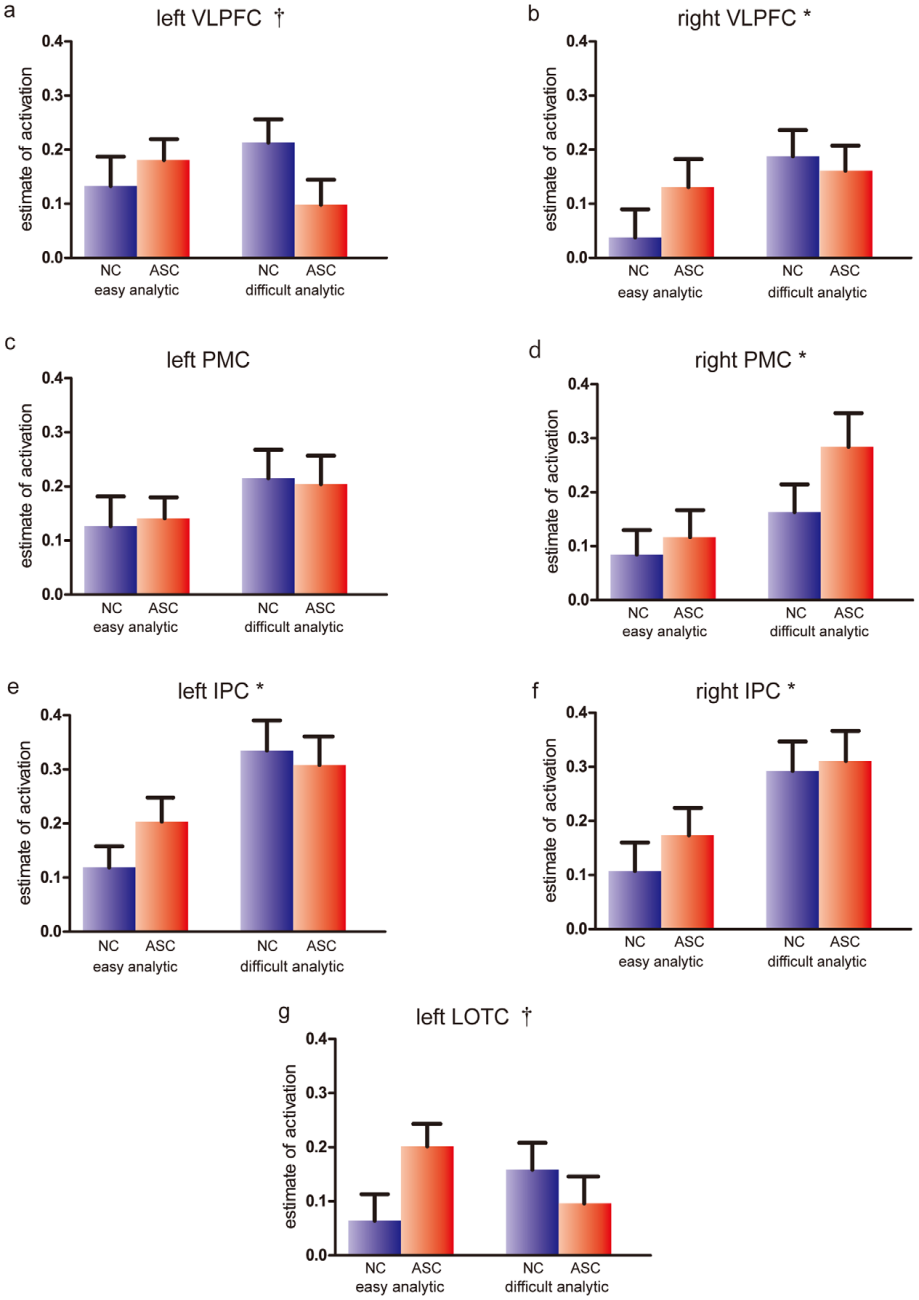
Region of interest (ROI) analysis of activation during the two analytic conditions. (a) Left ventrolateral prefrontal cortex (VLPFC), (b) right VLPFC, (c) left premotor cortex (PMC), (d) right PMC, (e) left inferior parietal cortex (IPC), (f) right IPC, and (g) left lateral occipitotemporal cortex (LOTC). Error bars indicate the standard error of mean. †: A significant interaction of Group (normal contorol/autism spectrum condition)×Condition (easy/difficult) *: A significant main effect of Condition.

To identify locations of activation foci unbiased for both groups, we first performed the contrast of analytic (easy and difficult) vs. baseline by combining NC and ASC. We found reliable activation bilaterally in the IPC, PMC, and VLPFC and in the left LOTC (Figure 3e, Table 5). This pattern of activation is consistent with previous studies using the RPM tasks [2], [11], [12], [14]. The left-lateralized activation of the LOTC is also consistent with a previous fMRI study that contrasted analytic with figural problems [7]. A three-way ANOVA (Group×Condition×Region) on estimates of activation of these seven regions revealed significant main effects of Condition (*F* = 11.49, *p* = 0.001) and Region (*F* = 6.12, *p* = 0.02). Follow-up two-factor ANOVA was then performed on each ROI (Figure 4). No regions showed a significant main effect of Group (all *p*>0.2). However, a significant main effect of Condition was found in the right VLPFC (*F* = 4.13, *p* = 0.04) and PMC (*F* = 8.89, *p* = 0.004) and bilateral IPC (*F* = 20.9, *p*<0.001 for left; *F* = 17.6, *p*<0.001 for right). We also found a significant interaction of Group×Condition in the left LOTC (*F* = 4.96, *p* = 0.03) and the left VLPFC (*F* = 5.98, *p* = 0.02). No other region showed a significant main effect of Condition (*p*>0.12) or interaction of Group×Condition (*p*>0.09).

**Table 5:** Significant activity during the analytic conditions (easy+difficult).

	Left					Right				
Region label	x	y	z	z value	Cluster size	x	y	z	z value	Cluster size
A: NC										
IPC	−44	−76	44	4.77	689	54	−70	34	4.63	400
VLPFC	−42	48	−10	3.69	130	34	34	44	3.42	140
PMC	−36	16	56	3.48	78					
B: ASC										
IPC	−48	−64	40	5.03	894					
PMC	−36	12	52	4.78	698	36	28	56	4.35	299
LOTC	−62	−28	−16	4.35	480					
VLPFC	−38	52	−2	3.91	114					
C: NC and ASC										
IPC/	−50	−68	44	6.62	2185	54	−62	46	5.84	1700
LOTC	−64	−28	−16	4.65						
PMC	−36	14	54	5.40	1301	38	28	54	4.66	1004
VLPFC	−42	48	−6	4.99	875	38	50	−10	4.42	291
Precuneus						4	−64	40	4.25	298

*p*<0.001, uncorrected for multiple comparison at the voxel level. Extent threshold: *k*>72 voxels.

Coordinates are in the MNI space.

NC: Normal Control, ASC: Autism Spectrum Disorder, IPC: inferior parietal cortex.

PMC: premotor cortex, VLPFC: ventrolateral prefrontal cortex, LOTC: lateral occipitotemporal cortex.

### Functional connectivity analysis

**Figure 5:**
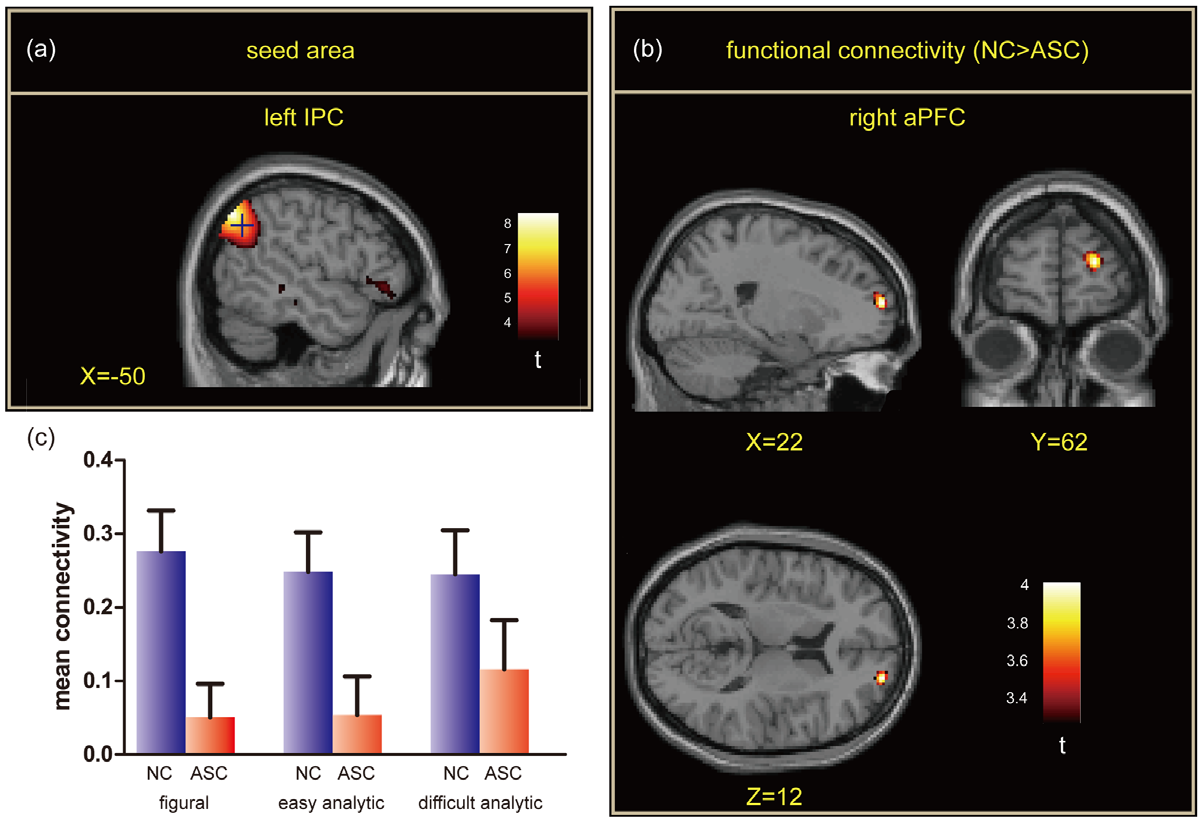
Group comparison of functional connectivity throughout all conditions. (a) The location of the seed area in the left inferior parietal cortex (IPC). The cross hair shows the focus of the seed area. (b) The right anterior prefrontal cortex (aPFC) showing reduced connectivity with the left IPC in autism spectrum condition (ASC) compared with normal control (NC). The peak of the group difference is shown in the MNI space. The cluster size was 109 voxels. *p*<0.001, uncorrected for multiple comparison at the voxel level. (c) Mean connectivity (Z value) in the right aPFC.

Among the regions in the frontoparietal network for fluid general intelligence, only analysis using the left IPC as the seed revealed a cluster of voxels with significant group difference (see Figure 5a for location of the seed). The significant voxel cluster was found in the right anterior prefrontal cortex (aPFC) showing reduced connectivity for ASC compared with NC (Figure 5b). Figure 5c shows the strength of functional connectivity with the left IPC during the figural, easy analytic, and difficult analytic conditions. A two-way ANOVA of Condition×Group revealed a significant main effect of Group (*F* = 7.06, *p* = 0.011), but no significant main effect of Condition or interaction (both *p*>0.20). These results show that activity coupling between the left IPC and right aPFC is reduced throughout the RSPM trials irrespective of distinction between figural and analytical items.

## Discussion

Using the RSPM task, the present fMRI study explored functional characteristics of the neural substrates of geometric reasoning in ASC. Our findings revealed that, among nodes of the brain network of fluid intelligence, ASC patients showed larger activation in the left LOTC during the easy analytic task than NC, whereas both groups showed comparable activation during the difficult analytic task. ROI analysis confirmed interaction of Group and Condition, indicating that while activity increased with task difficulty in NC, such modulation of activity was absent in ASC. Furthermore, functional connectivity analysis revealed that ASC showed significant reduction of activation coupling of the left IPC with aPFC in the right hemisphere. These results indicate that neural substrates of geometric analogical reasoning are functionally altered in ASC in terms of both localized responsiveness of the left LOTC and cortical interaction within the large-scale frontoparietal network of fluid intelligence.

### Enhanced activity of the left LOTC for analogical reasoning

In the analysis of activation magnitude, the left LOTC was the only region in the network of fluid intelligence that showed a significant group difference in both voxel- and ROI-based analyses. While NC showed increased activity with task difficulty, ASC showed an already-elevated activation level during the easy analytic condition without further enhancement of activation. Such altered relationship between activation magnitude and task difficulty has been reported in several clinical conditions. In previous studies that parametrically examined neural response for working memory load schizophrenia, the peak of the inverted U-shaped curve of the load-response function was shifted to a lower load in patients compared with normal controls [33]. A further hypothesis has been proposed, i.e., the curve of schizophrenia is flatter than that of controls reflecting a decreased ability to modulate neural response in accordance with memory load [34]. A similar shift of the activation peak was also shown in subjects with obsessive-compulsive disorder by a recent fMRI study using the parametric working memory paradigm [35]. These observations indicate that optimal cognitive load for engaging neural resources may be abnormal in certain psychiatric conditions. Similar to these observations, our finding suggests that in ASC, neural recruitment of the left LOTC for the geometric analogical reasoning complexity is altered, suggesting the possibility of either pathophysiological changes in this region or a different task strategy employed in geometric reasoning.

Although the exact roles of the left LOTC in fluid intelligence remain inconclusive, several previous studies have reported involvement of this region in analogical reasoning using geometric pictures [7], [14], [18], [19], [36]. Consistent with these reports, a recent fMRI study has demonstrated subdivision of the fluid intelligence network such that the left anterior LOC and VLPFC were sensitive to the factor of analogical reasoning, whereas the right lateralized frontoparietal network was influenced by rule complexity [16]. It is interesting that significant interaction of Group×Condition was found in both the left VLPFC and LOTC that comprises the anterior LOC in our analysis. The present results show that the two areas of analogical reasoning are easily engaged in response to analogical reasoning, but modulation of activity is less sensitive to task complexity in ASC. It is possible that ASC patients may lack flexibility in neural recruitment of these regions depending on task difficulty.

Increased activation of the left LOTC in the easy geometric analogical reasoning is also consistent with the proposal of an enhanced perceptual functioning (EPF) model of ASC [37]. The LOTC is the high-order area in the ventral visual pathway representing visual objects in abstract and multimodal forms [38], [39]. Our observation indicates that enhanced visual perception in ASC may be subserved by enhanced local responsiveness of the higher visual areas in addition to increased activation in the lower visual areas [17]. Given the abstract and multisensory nature of the LOTC representation, it is possible that functional enhancement of this area may contribute to the high level of abstraction of geometric objects necessary to detect regular relationships among multiple geometric objects.

### Altered functional connectivity for geometric reasoning

Functional connectivity analysis revealed significant reduction of activation coupling between the left IPC and right aPFC during geometric reasoning in ASC. This finding is in contrast to the observation that localized activation of the left LOTC was enhanced in ASC at least during the easy analytic condition. A number of previous fMRI studies have indicated that the right aPFC is crucial for higher-level cognition, including reasoning and integration of diverse information [40]–[42]. Furthermore, a recent fMRI study examined the development of functional connectivity in typically developing individuals and showed that pathways involving the right aPFC contributed the most to the prediction of brain maturation [43]. It is therefore likely that the functional development of the pathways involving this region is important for the typical development of the geometrical reasoning ability. Together with enhanced local activity in the left LOTC, our observations are consistent with the proposal that the ASC brain can be characterized by massive local connectivity and underdeveloped long-range connectivity [20]–[22]. Specifically, impairment of the inter-hemispheric connectivity in ASC has been indicated by abnormal size and microstructures of the corpus callosum [44], [45] and reduced functional connectivity between homologous areas [46]. Although it remains to be seen whether enhanced left LOTC activity and the long-range contralateral connectivity involving the right prefrontal cortex complement each other, our findings indicate altered involvements of these neuronal components in the network for fluid intelligence that may underlie their unexpectedly developed geometric reasoning ability and other uneven profiles of intelligence.

### Limitations and perspectives

The first aim of the study was to identify activation for analogical reasoning by comparing analytic with figural problems. In this design, potential group differences in activation for figural processing, if any, cannot be examined. The design was based on the analysis of cognitive components that both analytic and figural problems would involve figural processing, but only analytic problems would demand higher processes of analogical reasoning, such as the induction of abstract relations, strategy shifting and planning, goal management, and executive control of these processes [7]. Because we were primarily interested in those higher-level cognitive processes crucial for analogical reasoning rather than figural processing, we isolated processes for analogical reasoning by cognitive subtraction assuming the hierarchical relationship between the two classes of RSPM items. However, our approach does not exclude the possibility that activation during figural trials is different between NC and ASC subjects. Rather, our analysis of functional connectivity showed a significant group difference during figural blocks, which raises the need for future studies to include a lower-level control condition and to compare it with the figural condition. Therefore, the analysis of activation in the present study allows us to examine alternations of a subset of all the cognitive processes for geometrical reasoning in ASC.

Although the present study was motivated by the large discrepancy between RSPM and WAIS in ASC [5], such a discrepancy might only be seen in a subset in the autism spectrum population. Indeed, a recent study has reported that RPM advantage is significant only for ASC individuals with IQ scores of less than 85 but not for the population with higher IQs [47]. Because we designed the present experiment to minimize behavioral differences between groups in order to prioritize the interpretability of fMRI results [28], [29], the absence of group differences in the behavioral data collected during the fMRI session does not directly assess whether or not the patient population in the present study does show the RPM advantage. However, another recent study has replicated the RPM advantage over FIQ and PIQ for adults with Asperger's syndrome [24] who do not show the visuospatial peak in the WAIS subtest profile. Adult individuals with Asperger's syndrome are characterized by high IQs and significantly higher VIQ than PIQ [25]. We emphasize that the cognitive profile of the present ASC population was similar to that of Asperger's syndrome, suggesting that our ASC population is also subsumed under the range of the spectrum showing the RPM advantage.

To conclude, we found fMRI evidence that the neural system of geometric reasoning is functionally altered in high-functioning adults with ASC. The observations of an elevated level of activity in the left LOTC and a reduced activity coupling of the left IPC with the right aPFC support the view that the neural recruitment in ASC is biased toward the functionally specialized local regions rather than the distributed large-scale network. Such differential involvement of the brain system may help explain the uneven profile of neural substrates reflecting cognitive ability in ASC.
